# Analysis of the interaction of calcitriol with the disulfide isomerase ERp57

**DOI:** 10.1038/srep37957

**Published:** 2016-11-29

**Authors:** Elisa Gaucci, Domenico Raimondo, Caterina Grillo, Laura Cervoni, Fabio Altieri, Giulio Nittari, Margherita Eufemi, Silvia Chichiarelli

**Affiliations:** 1 Department of Biochemical Sciences “A. Rossi Fanelli”, Sapienza University of Rome, Piazzale Aldo Moro 5, 00185, Rome, Italy; 2 Stem Cell Lab - Department of Molecular Medicine - Sapienza Università di Roma, Viale Regina Elena 324, 00161, Rome, Italy; 3 Istituto Pasteur-Fondazione Cenci Bolognetti, Sapienza University of Rome, Piazzale Aldo Moro 5, 00185, Rome, Italy

## Abstract

Calcitriol, the active form of vitamin D_3_, can regulate the gene expression through the binding to the nuclear receptor VDR, but it can also display nongenomic actions, acting through a membrane-associated receptor, which has been discovered as the disulfide isomerase ERp57. The aim of our research is to identify the binding sites for calcitriol in ERp57 and to analyze their interaction. We first studied the interaction through bioinformatics and fluorimetric analyses. Subsequently, we focused on two protein mutants containing the predicted interaction domains with calcitriol: *abb’*-ERp57, containing the first three domains, and *a’*-ERp57, the fourth domain only. To consolidate the achievements we used the calorimetric approach to the whole protein and its mutants. Our results allow us to hypothesize that the interaction with the *a’* domain contributes to a greater extent than the other potential binding sites to the dissociation constant, calculated as a Kd of about 10^−9^ M.

The endoplasmic reticulum (ER) protein ERp57 is a member of the disulfide isomerase family and is involved in the folding and reshuffling of disulfide bonds in nascent glycoproteins, acting in cooperation with the lectins calreticulin and calnexin. All the disulfide isomerases share a thioredoxin fold and have catalytic and non-catalytic domains, called respectively *a*-type or *b*-type domains. ERp57 has four thioredoxin-like domains, with the *a* and *a’* catalytically active domains in the N- and C-termini. Most of ERp57 is located in the ER lumen, but unusual locations have been reported too, such as nucleus, cell membrane, cytosol and mitochondria^1^, even though the functions outside the ER remain elusive. STAT3, member of the signal transducers and activators of transcription (STAT) family, is a known interactor of ERp57 in the cytosol, cell membrane and nucleus, where the two proteins bind together to the C-reactive protein (CRP) gene promoter^2^. ERp57 may also directly bind DNA, as revealed by *in vitro*^3^ and *in vivo* studies^4^. ERp57 has been found to interact strongly with a number of small ligands, such as antibiotics^5^,^6^ and polyphenols^7^, as well as to macromolecules^8^,^9^,^10^.

ERp57 has been unexpectedly revealed as the membrane-associated receptor for calcitriol, the biologically active form of vitamin D_3_, responsible for the rapid nongenomic response to the hormone^11^.

The vitamin D_3_, which is formed in the skin after exposure to sunlight, needs two hydroxylation reactions to become the active form 1,25-dihydroxyvitamin D_3_, also known as calcitriol. Its mechanism of action is similar to other steroid hormones and involves the binding to the intracellular receptor VDR^12^. After this interaction, calcitriol/VDR heterodimerizes with the retinoid X receptor (RXR) and the heterodimer binds specific response elements, leading to either the activation or repression of gene transcription. The transcription process proceeds through the interaction of VDR with coactivators and with the transcription machinery^13^. In this way, calcitriol stimulates calcium and phosphate transport from intestine and kidney to the blood, but it has also anti-proliferative and pro-differentiating effects. In addition to the regulation of gene expression, calcitriol can exert rapid, nongenomic actions, which are performed by modulating the transmembrane transport of calcium and chloride ions and activating signal transduction pathways, such as those involving protein kinase C (PKC) and MAP kinases^14^. Among the MAP kinases, not only ERK1/2 is involved, but also ERK5, which participates in calcitriol-induced cell differentiation in acute myeloid leukemia^15^. More recently, it has been found that calcitriol inhibits Wnt/β-catenin signalling pathway in non-malignant murine colon cells^16^, while the inhibition of Hedgehog pathway is responsible for the anti-tumour effect of calcitriol in basal cell carcinoma^17^. The two proteins that could mediate the calcitriol-initiated signalling are VDR, which has been identified also in caveolae^18^, and a membrane-associated protein, which has been revealed as ERp57^11^. According to Doroudi *et al*.^19^, calcitriol interacts with ERp57 in caveolae, in complex with phospholipase A and caveolin 1 (Cav-1), leading then to the activation of phospholipase A2 (PLA2) and protein kinase C (PKC). In calcitriol-stimulated leukemia cells, ERp57 appears to be redistributed from plasma membrane and cytosol towards the nucleus, together with the transcription factor NFkB, and to act in the differentiation pathway^20^.

Recently some studies described a synergic action between ERp57 and VDR but not their direct interaction; in fact, the authors hypothesize a synergic action for the mineralization of pre-osteoblasts in 3D culture^21^ and for the Wnt5 calcium-dependent signaling mediated through Pdia3/ERp57, PLAA, and VDR^22^. However, another study describes a Pdia3/ERp57-mediated but VDR-independent vitamin D rapid response in osteoblasts with an increase in CaMKII (calcium/calmodulin-dependent protein kinase II) activity^23^.

Our studies on HeLa cells have shown that the stimulation with calcitriol causes the exit of ERp57 from the ER towards other cell compartments, such as the cytosol and the nucleus^24^. Currently, little is known about the role of ERp57 in non-ER localizations. It is possible to hypothesize the involvement in signal transduction processes in response to different extracellular stimuli and the trafficking through the cell. In fact, early studies on ERp57 have misidentified this protein as phospholipase C alpha, but they have shown that ERp57 is able to interact with the angiotensin II receptor^25^ and the vasopressin receptor^26^. Moreover, its association with the angiotensin receptor is likely to possess functional significance, as suggested by its phosphorylation following angiotensin binding^27^. Zhu *et al*. have been reported a full description of the interaction of ERp57 with the all-trans retinoic acid receptor α in Sertoli cells. ERp57 is associated with the receptor in the cytosol and is required for the transport of the ligand-receptor complex into the nucleus, and subsequently into the ER to allow the receptor degradation by the ERAD (ER-associated protein degradation). The role of ERp57 in the receptor activity has been ascribed to the conformational changes of the receptor, in order to facilitate the binding of the ligand.^28^. Sehgal and collaborators found STAT3 in the lipid raft fraction of cell membrane, associated with ERp57^29^. The involvement of ERp57 in signal transduction processes has been demonstrated for STAT3-involving pathways as described above^2^. Moreover, it has been found that the ERp57 silencing affects the internalization and phosphorylation of the EGF receptor (EGFR) after EGF binding^30^.

The identification of the calcitriol binding site in the ERp57 structure can help to elucidate the role of ERp57 in its mechanism of action, in order to clarify the intracellular trafficking of the complex. In this context, our investigation workflow has involved different approaches. We have performed a bioinformatic analysis of the ERp57 structure and of the interaction with calcitriol, as well as a spectrofluorimetric analysis of their interaction. After these results, we have focused our study on specific deletion mutants of the protein. The mutants have been chosen on the basis of the *in silico* results. In particular, one mutant is composed by the first three domains, which are the catalytically active *a* domain and two adjacent *b*-type domains (*abb’*-ERp57), while the other one is the fourth domain only (*a’-ERp57)*. Moreover, for the *abb’*-ERp57 mutant we have refined a specific expression and purification protocol. In order to consolidate the data obtained from the bioinformatic and fluorimetric analyses, we performed the isothermal titration calorimetry of the whole protein and its two mutants.

## Results

### ERp57-calcitriol interaction prediction

**Figure 1:**
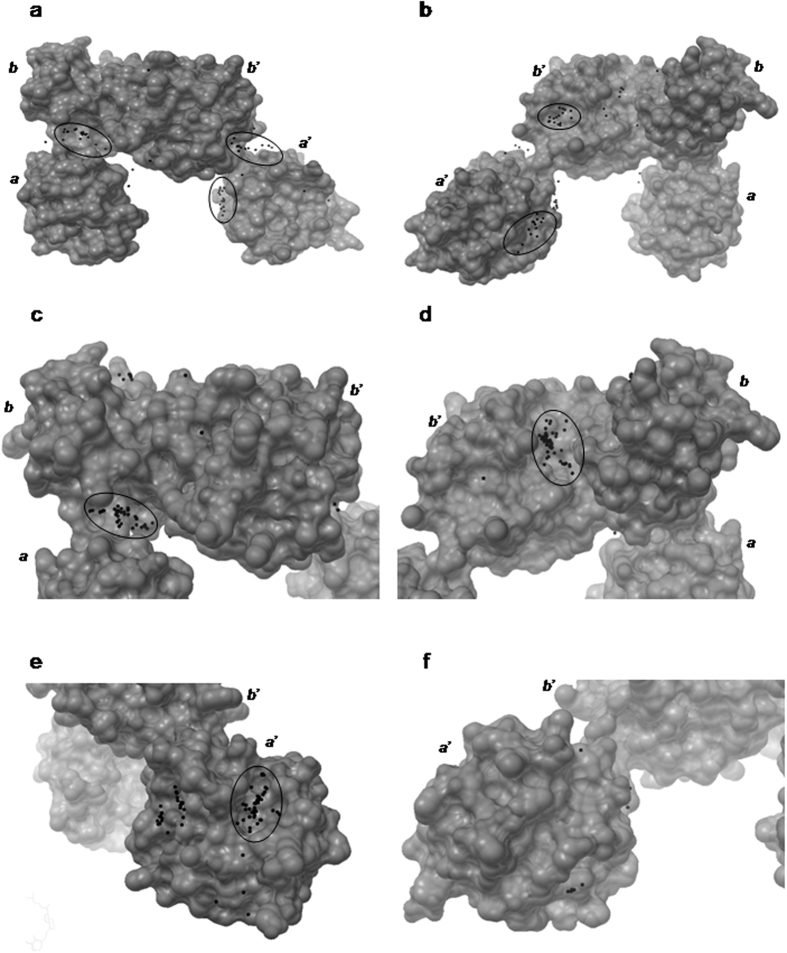
Docking analysis performed on the entire ERp57 protein (**a,b**), or at the level of the *bb’* domains (**c,d**) or *a’* domain (**e,f**). The protein is depicted in grey as surface. Each docked conformation is represented by a sphere placed at the average position of the coordinates of all the atoms in that conformation. The radii of the spheres are 0.3 Å.

Blind docking experiments, i.e. a single docking experiment carried out on the whole ERp57 protein surface, were performed in order to identify putative druggable cavities that calcitriol can explore. This approach for finding the putative binding site has already been successfully applied to other biological targets^31^,^32^,^33^. Binding modes of calcitriol molecule with the most favorable energies were evaluated and clustered. Results obtained clearly show that *a’* and *bb’* domains of ERp57 are preferred by calcitriol (see Fig. 1a–f in which each docked conformation has been represented as a sphere whose center is at the average position of all the atoms in that conformation). These data suggest that it is very likely that these regions correspond to the binding portions explored by the calcitriol molecule. Two out of three regions predicted by blind docking approach were also identified as potentially establishing favorable interactions with small ligands by computational solvent mapping analysis^34^ (see Material and Methods), thus improving the reliability of our hypothesis that these regions are compatible with calcitriol binding. In fact, as shown in Fig. S7, the organic probes clusters are all in the *b*, *b’* and *a’* domains. Therefore, in order to refine our results, we performed focused docking experiment increasing the number of energy evaluations and varying the docking box resolution. The search space was restricted to the vicinity of the binding sites both predicted by blind docking and confirmed by the FTsite program on *a’*, *b* and *b’* domains, discarding *a* domain.

**Figure 2:**
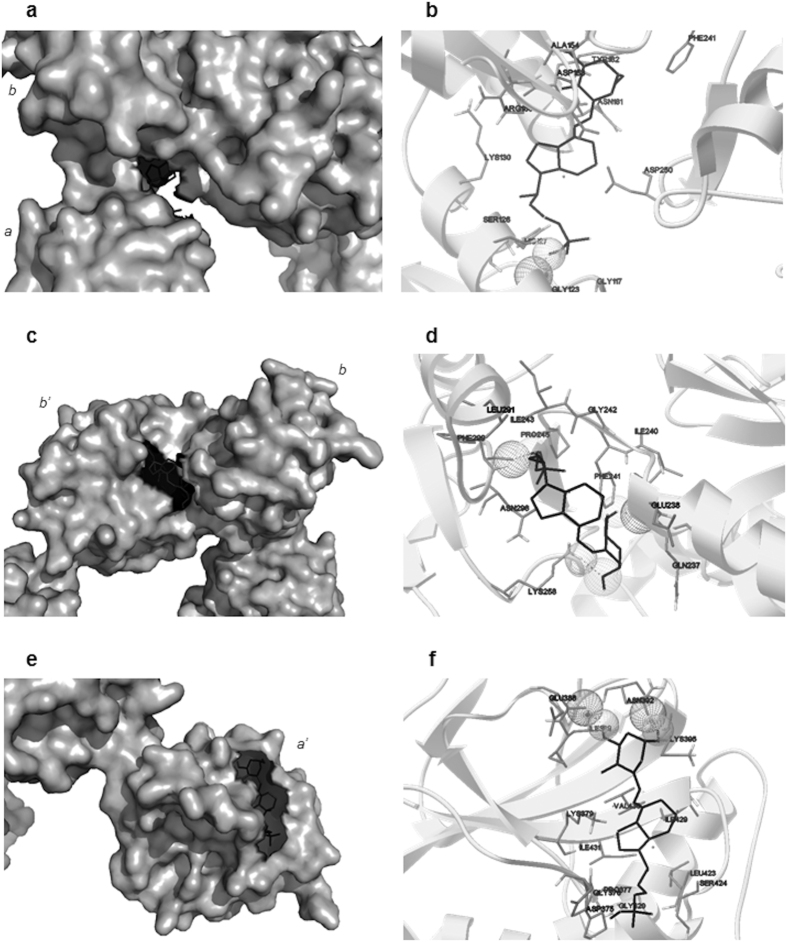
Docking analysis on the cavities located in the *b* domain of ERp57 (**a,b**), between the *b* and *b’* domains (**c,d**), on the *a’* domain (**e,f**). (**a,c,e**) The lowest energy conformer of the most populated cluster is shown in black sticks on the surface of the protein. (**b,d,f**) detail of the interactions between the lowest energy conformers and the surrounding residues. The hydrogen bonds are shown in wireframe and with dashed lines. The protein is depicted as cartoon.

Focused docking experiment consisted of three independent runs, with the docking box centered on the predicted druggable hot spots. Figure 2 shows results of the dockings experiments by exploring cavities in the *b* domain (Fig. 2a,b), between the *b* and *b’* domains (Fig. 2c,d) and in the *a’* domain (Fig. 2e,f). We selected the lowest energy conformer belonging to the most populated cluster as the most likely calcitriol pose. It can be noticed that in the cavity in the *bb’* domains calcitriol is involved in two hydrogen bonds with Glu238 and Lys258, and in the *a’* domain with Glu388 and Asn392.

Collectively, these results strongly support hypothesis that calcitriol binding region can be formed by well defined pockets in the *bb’* and *a’* domains, thus putting the basis for a structural interpretation of the binding of the calcitriol molecule to the ERp57 protein surface.

### Purification of recombinant mutant abb’-ERp57

To analyse the interaction between calcitriol and the *abb′*-ERp57 mutant, we expressed and purified for the first time the recombinant protein consisting of *abb′* domains without GST-fusion, and tested its binding activity (see Material and Methods).

SDS–PAGE of different protein fractions during purification of *abb’*-ERp57 mutant are shown in Fig. S1 and Fig. S2 (see Supplementary data).

### Calcitriol interacts with ERp57 and its deletion mutants – fluorimetric assays

We have conducted *in vitro* studies with the aim of determining the affinity constant of the interaction between ERp57 and calcitriol. Fluorimetric assays were performed to measure the variation of the protein intrinsic fluorescence, due to three triptophane residues in the ERp57 sequence, after adding increasing concentrations of calcitriol. These assays were performed both on oxidized and reduced protein. In the former case, the recombinant ERp57 was oxidized with H_2_O_2_, and the binding was analyzed at the spectrofluorimeter, by measuring the emission at 336 nm wavelength. There was a quenching of the fluorescence emission of the protein by increasing concentrations of the ligand, in a large excess, but unfortunately the formation of both monomeric and dimeric species in solution, due to both intra and intermolecular disulfide bridges, did not enable us from deriving the dissociation constant.

In the case of totally reduced rERp57, with the use of TCEP as reducing agent, the affinity constant could be obtained, since only the monomeric form is possible.

Considering the highly hydrophobic nature of calcitriol, another fluorimetric assay, in which the ligand was not in large excess but was in nearly equimolar concentration with the protein in the reduced form, was performed to avoid the possible adsorption of calcitriol to the chamber of the quartz fluorimeter cuvette. The protein was finally saturated with an excess of ligand.

In Fig. S3, the graph shows a plot of the fluorimetric data, in which the variation of the intrinsic fluorescence of the protein is plotted as a function of the ligand concentration. The graphical display of the data allowed the determination of the dissociation constant, which was calculated with the following formula, according to Cogan *et al*.^35^.


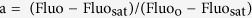



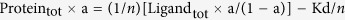


where Fluo_sat_ is referred to the fluorescence of the protein totally saturated with calcitriol, while Fluo_0_ to that of the reduced rERp57 in absence of the ligand.

Three independent experiments were performed and the mean dissociation constant, derived from the equation assuming y equal to zero, was calculated as 10^−9^ M, comparable with that of the nuclear VDR. *n*, which corresponds to the number of ligand molecules bound to one single protein molecule, was nearly two, consistent with the hypothesis of two binding sites in the ERp57 protein in the reduced form.

Three independent experiments were performed, also, with ERp57 mutants (*abb’*-ERp57 and *a’*-ERp57).

The data are consistent with a direct interaction between *a’*-ERp57 and calcitriol (Fig. S4); this evidence could be justified by a site-specific interaction of calcitriol with the *a’* domain with a Kd ∼10^−8^ M and a stoichiometry of ± 1.2 for the number of ligands bound.

On the other hand, the data obtained for the *abb’*-ERp57 mutant (Fig. S5) were of difficult interpretation and overall are not consistent with a line that brings out a direct interaction between the mutant and calcitriol. These unpredictable data may be due to the fact that the two triptophane residues in the *abb’*-ERp57 mutant could be partially quenched in the folding of the protein without the fourth *a’* domain.

### Calcitriol interacts with ERp57 and its deletion mutants – calorimetric assays

**Figure 3:**
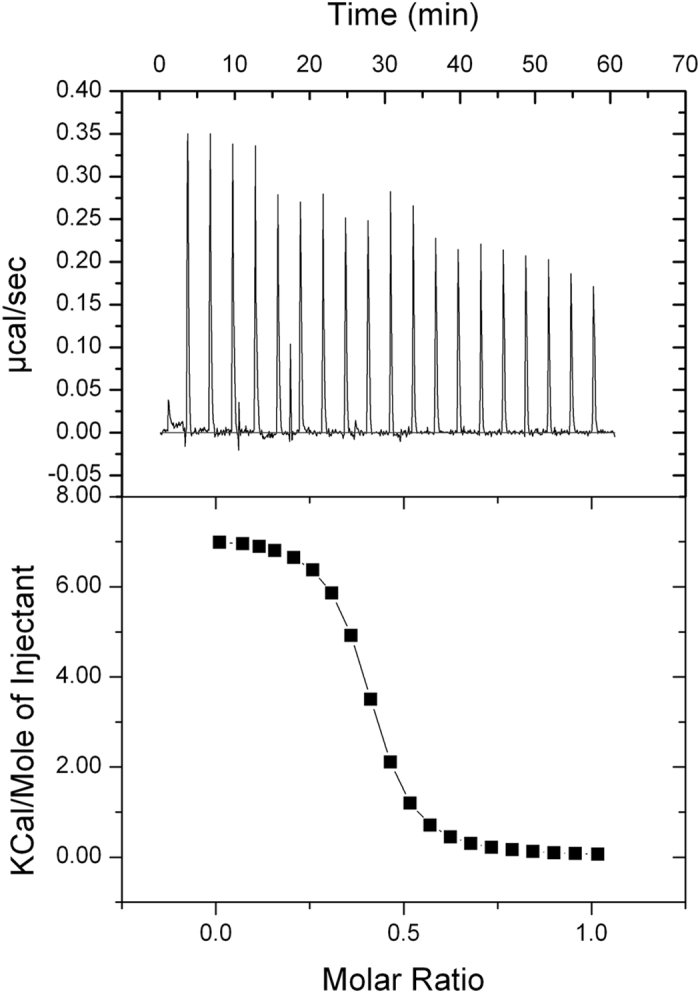
Isothermal Titration Calorimetry of *abb’*-ERp57 (20 μM) with calcitriol (100 μM). Both the protein and the ligand were in the same buffer (see Materials and Methods). (Upper panel) heat evolved upon injection of calcitriol into *abb’*-ERp57, plotted as a function of injection order. (Lower panel) Integrated heats of reaction plotted against the molar ratio of total ligand concentration to total protein concentration. The solid line shows the best fit to the data for n = 0.394 (±0.00721) sites/mole, Ka = 5.98 (±1.4) × 10^6^ M^−1^, ΔH = 7.140 ± 0.183 × 10^3^ kcal/mol.

**Figure 4:**
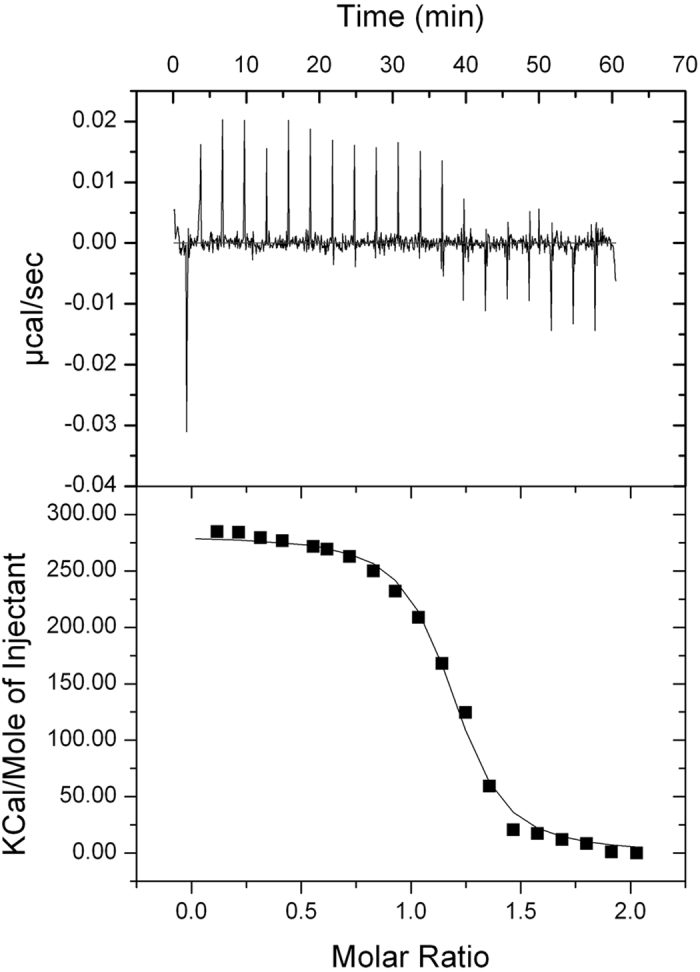
Isothermal Titration Calorimetry of *a*′-ERp57 (0.05 μM) with calcitriol (0.5 μM). Both the protein and the ligand were in the same buffer (see Materials and Methods). (Upper panel) heat evolved upon injection of calcitriol into *a’*-ERp57, plotted as a function of injection order. (Lower panel) Integrated heats of reaction plotted against the molar ratio of total ligand concentration to total protein concentration. The solid line shows the best fit to the data, according to a model that assumes a single set of identical sites, for n = 1.15 (±0.00892) sites/mole, Ka = 1.68 (±0.231) × 10^9^ M^−1^, ΔH = 2.813 ± 0.032 10^5^ kcal/mol.

In order to identify the domain(s) of ERp57 responsible for the interaction, the two deletion mutants of the protein were tested by Isothermal Titration Microcalorimetry (ITC) (Fig. 3 for *abb’*-ERp57 and Fig. 4 for *a’*-ERp57). Before the assay, the mutants were reduced with TCEP.

The microcalorimetric titration curve fitted a possible model of interaction and the same model was matched with the results of fluorescence measurements. A value of Kd of ∼10^−9^ M was obtained, consistent with the fluorimetric results.

**Figure 5:**
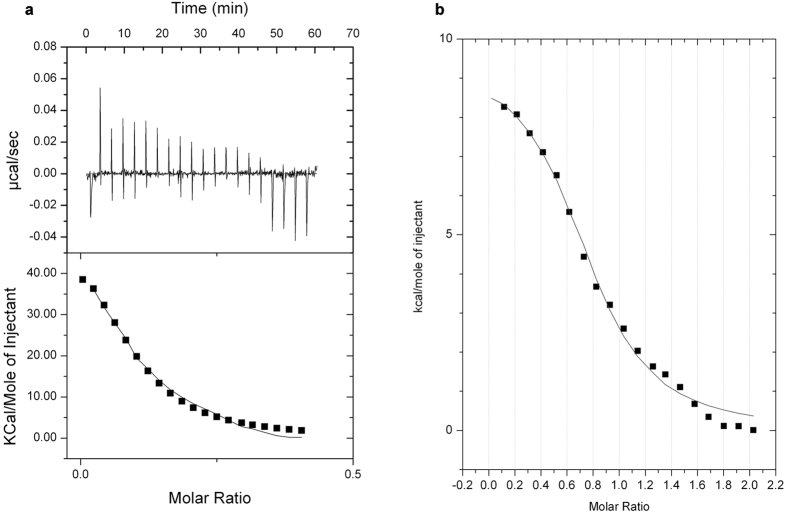
(**a**) Isothermal Titration Calorimetry of ERp57 (5 μM)/Silibinin (50 μM) with calcitriol (10 μM). Both the protein and the ligands were in the same buffer (see Materials and Methods). (Upper panel) heat evolved upon injection of calcitriol into ERp57/Silibinin, plotted as a function of injection order. (Lower panel) Integrated heats of reaction plotted against the molar ratio of total ligand concentration to total protein concentration. The solid line shows the best fit to the data, according to a model that assumes a single set of identical sites (for Kd see Table 1) (**b**) Isothermal Titration Calorimetry of ERp57 (5 μM) with Silibinin (50 μM). The integrated heat of reaction is plotted against the molar ratio of total ligand concentration to total protein concentration. The solid line shows the best fit to the data for n = 0.776 (±0.0183) sites/mole, Ka = 2.07 (±0.273) × 10^6^ M^−1^, ΔH = 9.565 ± 0.312 10^3^ kcal/mol.

The thermodynamic data were processed as described above. The values of ΔH were measured for each titration and the system also gave information on the change in entropy (ΔS). The binding free energy (ΔG) and the dissociation constant (Kd) were calculated from the experimentally determined values of ΔH and Ka, using eqs 1 and 2 (see Materials and Methods). The *in vitro* binding of calcitriol to ERp57 was studied also by competition ligand binding by displacement ITC (Fig. 5a,b).

The apparent binding constant, in the competition experiments, depends on the concentration of free inhibitor, which changes during the experiment. The binding constant was calculated as described in Sigurskjold^36^ (see Materials and Methods).

The calorimetric data are reported in Table 1.

The evidences indicated that the binding to each mutant was entropically driven. The value of TΔS is 295 kcal/mol and 17.2 kcal/mol for the *a’*-ERp57 and *abb’*-ERp57 mutants respectively; the ΔH values are 283 kcal/mol and 8.00 kcal/mol. Moreover, we observe that calcitriol binds ERp57 with a ratio approximately of 1:1 for the *a’*-ERp57 mutant.

## Discussion

The disulfide isomerase protein ERp57, which mainly resides in the ER, is known to participate in a number of well-studied processes, such as the quality control of newly synthesized glycoproteins^37^ and in the assembly of MHC class I. In other subcellular compartments, where it is present in small amount, it is involved in a variety of mostly unexplored processes.

In the present research, the interaction of ERp57 with calcitriol has been explored. The biologically active form of vitamin D_3_ can exert its function of regulation of gene expression through the binding to the nuclear receptor VDR, but it is also able to act through a membrane-associated receptor, displaying a wide variety of rapid, nongenomic actions, such as the rapid activation of signalling cascades. The membrane associated receptor was revealed to be ERp57^11^, which was originally called membrane-associated rapid response steroid binding protein (1,25D3-MARRS). Furthermore, it has been demonstrated that the ERp57-calcitriol complex, which has been found in the matrix vesicles of chondrocytes, is implicated in the reorganization of extracellular matrix^38^. In this context, the expression of ERp57 was found to be decreased in the osteogenic differentiation of mouse embryoid bodies, contrarily to VDR, leading to the hypothesis of a differential expression of the two proteins at diverse stages of cell commitment^39^. In this work is shown, for the first time, the direct interaction between ERp57 and calcitriol using the purified recombinant protein and its deletion mutants.

To date, a lot of studies have investigated the function of the interaction of ERp57 with calcitriol^40^,^41^,^42^,^43^,^44^,^45^. Moreover, in a review on vitamin D analogues as potential therapeutics in melanoma, the 1,25-dihydroxy-lumisterol, locked in the 6-cis configuration, was cited as a vitamin D analogue which activates the rapid-response pathways and competes with 1α,25-dihydroxyvitamin D_3_ for MARRS receptor binding, but not for VDR binding^43^. In addition, Tohda *et al*. in 2012^46^ have presented a docking simulation between ERp57/1,25D3-MARRS and diosgenin which is very similar in structure to calcitriol; in particular in this study they found that the exogenous stimulator diosgenin activates the 1,25D3-MARRS pathway, which may be a very critical signalling target for anti-AD (Alzheimer’s disease) therapy.

Our study analyses in details, for the first time, the direct interaction between calcitriol and ERp57 by means of *in vitro* techniques and we have, also, hypothesized the probable interaction sites.

By means of fluorimetric assays (Fig. S3), we have derived the dissociation constant of the complex between recombinant ERp57, in the reduced form, and calcitriol, which was calculated as 10^−9^ M. Above all, our data confirm the Kd value of this interaction, indicated previously in the literature^47^.

The first step of our bioinformatic analysis has been the detection of pockets or cavities in the ERp57 structure (Fig. S6 and Table S1). Among the available programs, FTSite^34^ has been chosen. The prediction algorithm relies on the experimental evidence that small organic molecules are able to bind ligand binding sites. In this way we detected three possible binding sites in the ERp57 structure: one bigger cavity between the *b* and *b’* domains, and two smaller cavities, very close to each other, buried in the *b* domain. Then, the FTMap server^48^ has been used, to sample the entire protein surface and identify “hot spots”, which can be defined as locations in the protein that contribute to the ligand binding free energy. As shown in Fig. S7, the organic probes cluster are all in the *b* and *b’* domains, some of them overlapping with the cavities detected with FTSite, with the exception of one, which is located in the *a’* domain. The normal mode analysis, to study the dynamic properties especially in the hinge regions, did not show a substantial difference in the three cavities, while the *a’* domain was revealed as very flexible (Fig. S8 and Table S2).

Subsequently, docking analysis of calcitriol into the X-ray crystal structure of ERp57 was performed with Autodock, to investigate the direct interaction between the ligand and the protein. After a first blind docking (Fig. 1), analyses were performed centring the grid box in the *bb’* and *a’* domains. Regarding the *bb’* domains, the conformations are mainly distributed in the cavities identified by FTSite, while in the *a’* domain, the majority is found in the same region already identified by FTMap. Consequently, a more detailed analysis, increasing the energy evaluations, was performed in these cavities (Fig. 2). In all cases, calcitriol is able to establish not only hydrophobic interactions, but also hydrogen bonds, both as donor and acceptor, due to its three alcoholic moieties. In addition to the main chain, the residues that could form hydrogen bonds with calcitriol are Glu238 and Lys258 in *bb’*, Glu388 and Asn392 in *a’* domain. All clusterings were performed at 2.0 Å RMSD tolerance. The results of the docking in the *a’* domain were then re-clustered at 10 Å, to see if it was possible to obtain a single conformation (Fig. S9). The clusters were reduced to 2, from 11 deriving from the 2.0 Å clustering. In the second cluster (16 poses out of 100), the A ring of calcitriol points at the α helix of ERp57, in the first one (84 poses out of 100) not. In both cases, the two fused rings are partially superimposed to the hot spot found by FTMap. The *in silico* study has therefore highlighted the potential binding sites, present on the a’ domain, and *bb’* domains.

In the present work we also described in detail, for the first time, the protocol of expression and purification of the *abb’*-ERp57 mutant, which has been used for the *in vitro* binding studies with calcitriol.

The spectrofluorimetric and calorimetric binding experiments have confirmed the direct interaction between ERp57 and calcitriol and also defined the Kd of the interaction. In particular, the analysis of the *in vitro* binding between the purified recombinant protein and calcitriol, conducted by spectrofluorimetry, has shown a Kd rather low, ≈10^−9^ M, hence a rather high affinity, comparable with that of calcitriol against its canonical nuclear receptor (VDR). The spectrofluorimetric assays performed on the two mutants of the protein (*abb’*-ERp57 and *a’*-ERp57), did not allow us to make assumptions about the interaction, probably because of the low sensitivity of the method. The spectrofluorimetric analysis is based on the intrinsec fluorescence of tryptophans, on the other hand this value depends on the position of the residues inside the protein. In the *abb’*-ERp57 mutant, the tryptophan residue is localized in a hydrophobic pocket and although it can potentially be part of the binding site, this specific localization could affect the fluorescence variation, which may be minimal and therefore could not be well detected.

For what concerns the spectrofluorimetric data of the calcitriol/*a’*-ERp57 interaction, we could hypothesize a direct interaction with a Kd of ≈10^−8^ M. This result, however, presents high variability, as highlighted by the standard deviation of the single points of the correlation graph. In this case, also, the low sensitivity of the method may have contributed to the experimental variability, in fact the *a’*-ERp57 mutant has only one triptophane residue.

The binding experiments between ERp57, or its mutants, and calcitriol were also repeated by titration calorimetry. Through this method, which is able to detect enthalpy and heat changes during a reaction, a very strong interaction with the ligand has been put in evidence. In particular, it has been possible to show an *abb’*-ERp57-calcitriol interaction with a Kd of ≈10^−6^ M, which was not possible to derive from the fluorimetric data. The calorimetric analysis with the *a’*-ERp57 mutant and calcitriol has highlighted a Kd constant of ≈10^−9^ M. This indicates the presence of a strong interaction site, validated by the potential binding site highlighted by the *in silico* study; more than ever these data demonstrate for the first time that the ERp57 protein structure contains a domain that interacts directly with calcitriol.

In order to study the interaction of ERp57 with calcitriol by titration calorimetry, we proceeded with competition experiments. We used the displacement titration calorimetry technique because our data, obtained by fluorimetric assays, indicate that the binding constant between ERp57 and calcitriol should be near 10^−8^ M. Usually the direct measurement of very large binding constants would require so low concentrations that the signals become too small; this problem can be solved by using the displacement titration calorimetry, in which a less strongly bound ligand is competitively inhibiting the binding of the stronger ligand. To perform this kind of experiment we chose silibinin as a competitor, because it has an affinity of at least two orders of magnitude lower compared to calcitriol^49^. Thus, it was found that the constant calculated with the fluorimetric data was compatible and confirmed by the calorimetric data. The results of the displacement titration calorimetry technique, using ERp57/silibin/calcitriol, show that the affinity of the ligand corresponds to the interaction between calcitriol and the *a’*-domain alone. In a previous work, we have found that silibinin binds to the *aa’* domains^49^ and now the results presented here substantiate that the interaction site of calcitriol and silibinin is the same.

On the basis of these results, we can hypothesize that ERp57 and calcitriol interact directly and their Kd is about 10^−9^ M. In addition, these data recognize the *a’*-ERp57 domain as the site with the highest affinity, contributing to a greater extent than the other potential binding sites to the dissociation constant.

The results obtained by fluorescence measurements and the titration curve obtained by microcalorimetry indicate a binding reaction reaching an equilibrium.

The evidences performed by spectrofluorimetry experiments with FITC-Insulin^5^, show that there is not any alteration of the oxidoreductase activity of protein in the presence of calcitriol (Fig. S10 and Table S3).

The ERp57 domains involved in different interactions have been identified, i.e. calreticulin binds the *bb’* domains^50^ and vancomycin may hinder the interaction between calreticulin/ERp57^6^. Other proteins interact specifically with *a* and/or *a’*, such as REF-1/APE^9^ and also tapasin^51^. Moreover, different molecules interact with the same domains, i.e. DNA interacts with the *a’* domain^9^ and silibinin binds to the *a/a’* domains^49^.

The interaction of ERp57 with ligands such as silibinin^49^, the EGF receptor^30^ and calcitriol could induce the internalization of the protein. This event is the beginning, presumably, of specific processes of the signal transduction that could be deeply studied in order to understand more clearly the roles of ERp57 on the plasma membrane. Recently it was shown that the disruption of vitamin D/ERp57 pathway mimics amyloid pathology^52^; moreover Sugimoto *et al*.^53^ demonstrate that Denosomin-Vitamin D3 hybrids, used as anti-Alzheimer’s disease agents, exhibit nerve re-extension activity in Aβ-damaged neurons via the ERp57 (1,25D3-MARRS) pathway. Considering also the role of ERp57 in important cellular functions and the promising clinical use of vitamin D analogues in prevention or therapy in several types of malignancies^54^, the interaction between these two molecules is of sure interest and would need further investigations in the cellular context.

## Materials and Methods

### Cloning, expression, and purification of recombinant ERp57 and deletion mutants

Human recombinant ERp57 was cloned and expressed in E. coli BL21 strain using the expression vector pET21 (Novagen) as described previously^7^. The recombinant protein was purified by ammonium sulphate fractionation and chromatography steps using a procedure similar to the one employed for the purification of pig liver ERp57^9^. Also the *a’*-ERp57 mutant was obtained as illustrated previously^7^.

For the preparation of *abb’*-ERp57 mutant, a plasmid pGEX-2T vector containing the deletion mutant of human *abb’*-ERp57 fused to the glutathione S-transferase protein (GST), was used. The coding sequence of *abb’*-ERp57 was extracted from pGEX-2T vector by means of a specific restriction reaction (BamHI and EcoRI, Thermo Scientific). The same digestion was conducted on pET21a vector. The restriction fragments with the *abb’*-ERp57 coding sequence, without GST-protein, were purified with the Gel Extraction Kit (Qiagen) and then ligated into BamHI and EcoRI sites of a pET21a vector (LigaseT4, Thermo Scientific). Cloning was performed in DH5α E. coli by standard procedures^55^, while the protein was expressed in BL21 E. coli. The transformed cells were grown in 2YT medium containing 0.03 mg/ml ampicillin at 37 °C with shaking until the A600 reached 0.6–0.8 OD and then induced with 0.8 mM IPTG at 16 C overnight. Cells were harvested by centrifugation and resuspended in NEN buffer (20 mM Tris–HCl, pH 8.0, 100 mM NaCl, and 0.5 mM EDTA) containing 0.25% Triton X-100, 5 mM DTT and 0.2 mM PMSF. Cell suspension was lysed by sonication (Ultrasonic homogenizer UP100H) and cleared by centrifugation at 12,000 g for 10 min at 4 °C. The supernatant was fractionated by ammonium sulphate precipitation. The sonicated supernatant was incubated 2 h at 4 °C with 50% ammonium sulphate and then centrifuged at 12,000 g for 15 min; the resulting supernatant was added of 75% ammonium sulfate and incubated 2 h at 4 °C. After a centrifugation at the same speed, the pellet (with deletion mutant) was dissolved in 20 mM Tris-HCl, pH 8.0, 20 mM NaCl and dialyzed against the same buffer. Proteins were loaded onto a heparin column (Affi-Prep Heparin, Bio-Rad) and eluted with 15 volumes of a linear 40–1,000 mM NaCl gradient in 20 mM Tris-HCl, pH 8.0. Fractions containing the recombinant protein were pooled and dialyzed against 10 mM Tris-HCl, pH 8.0, 10 mM NaCl. The protein was further purified on a heparin column using a narrow NaCl gradient, dialyzed, and finally concentrated by using a Vivaspin concentrator (VivaScience).

### FITC-insulin reduction assay

Bovine insulin (Sigma-Aldrich) was labeled with fluorescein (Sigma-Aldrich) as described in Heuck and Wolosiuk^56^. FITC-insulin emission intensity was followed at 519 nm for 30 min at 25 °C, setting the excitation wavelength at 495 nm. For each reaction, 0.7 μM FITC-insulin was used, in a final volume of 2 ml. The baseline was derived from the addition of 10 μM DTT, in 50 mM Tris-HCl, pH 8.0, 1 mM EDTA. Subsequently, fluorescence enhancement was obtained by adding 0.1 μM ERp57. Calcitriol was then used at the following concentrations: 0.1, 1, 5 and 10 μM.

### Fluorescence quenching

Human recombinant ERp57, prepared as previously described, was oxidized for 1 h with 0.2 mM H_2_O_2_, and then extensively dialyzed with 20 mM Tris-HCl, pH 8.0, 20 mM NaCl. Alternatively, rERp57 was reduced with 1.25 μM TCEP (tris-(2-carboxyethyl)-phosphine) for 20 min at room temperature. The binding was measured by adding increasing concentrations of calcitriol (from 0.41 μM to 2.4 μM final concentration) to a 34 nM solution of oxidized or reduced rERp57, as described above, in 10 mM Tris-HCl, pH 8.0. The fluorescence of the protein was analyzed in a spectrofluorimeter (FluoroMax, Spex), thermostated at 25 °C, with an excitation wavelength of 280 nm and an emission of 336 nm. In another experiment, nearly equimolar concentrations of ligand were used, from 20 nM to 70 nM, and the protein was finally saturated with 2.4 μM calcitriol.

The experiment with mutants (*abb’*-ERp57and *a’*-ERp57) was performed using 1.5 μM solution of reduced ERp57-mutants and increasing concentrations of calcitriol from 0.9 μM to 3.105 μM final concentration.

Finally 20 μM calcitriol was added as saturation concentration.

### Calorimetric assay

ITC experiments were performed at 25 °C using a MicroCal ITC200 microcalorimeter (MicroCal Inc., Northampton, MA, USA). ERp57 and its deletion mutants were extensively dialyzed against the buffer of choice (0.2 to 1 mM NaCl; 20 mM Tris-HCl, pH 8.0) with Amicon Ultra filters, and the final exchange buffer was then used to dilute the silibinin stock solution (5 mM in DMSO) and the calcitriol stock solution (12 mM in ethanol and the percentage of the ethanol was below 0.84%); the DMSO was added to the protein solution at the same percentage of the ligand solution (below 1%). Samples were centrifuged before the experiments to eliminate possible aggregates. Protein and ligand solutions were degassed before use. Titrations were performed at 25 °C. The protein solution was placed in the sample cell, and each ligand solution was loaded into the syringe injector. The titrations involved 19 injections of 2 μL at 180 s intervals. The syringe stirring speed was set at 1,000 rpm. The reaction was very fast, as shown by the immediate appearance of an endothermic sharp peak following the addition of ligand to the protein and its deletion mutants solution in the microcalorimetric titration experiment. Reference titrations of ligand into buffer were used to correct for heats of dilution. The thermodynamic data were processed with Origin 7.0 software provided by MicroCal. The values of ΔH were measured for each titration, and fitting the binding isotherms with a one-site binding model yielded the values of the association constant (Ka). The system also gave information of the change in entropy (ΔS). The binding free energy (ΔG) and dissociation constant (Kd) were calculated from the experimentally determined values of ΔH and Ka, using eqs 1 and 2:


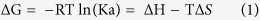



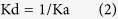


where R is the gas constant (1.987 cal•mol−1•K−1), and T is the working temperature (298 K).

The *in vitro* binding of calcitriol and ERp57 was studied also by competition ligand binding by displacement isothermal titration calorimetry. The apparent binding constant, in the competition experiments, depends on the concentration of free inhibitor, which changes during the experiment^36^.


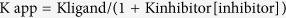



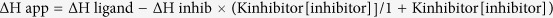


[Ligand:calcitriol, Inhibitor: silibinin]

In Table 2 the concentrations of ERp57 and its mutants are reported with the amounts of the ligand.

### Bioinformatics analysis

The FTSite server (http://ftsite.bu.edu)^34^ was used for binding site prediction on the experimental structure of ERp57. The strategy of FTSite consists in exploring the potential interactions of the surface regions of a protein with 16 small organic molecules, which vary in size and shape, and it has been shown to be effective in detecting ‘hot spots’ involved in binding to drug-size ligands Blind docking experiments were performed using AutoDock4.2 software^57^. We performed blind docking in order to find the binding sites of calcitriol onto ERp57 without any prior knowledge of its location. The initial coordinates of ERp57 have been obtained from the 2.6 Å resolution structure of tapasin-ERp57 heterodimer^51^ (PDB code: 3F8U). For the study presented here, we selected the coordinates of chain A, containing the residues 25–493 of ERp57 with the exclusion of the N-terminal signal peptide and of the residues 494–501 at the C-terminus. Input coordinates of calcitriol were extracted from the crystal structure of the nuclear receptor for vitamin d (VDR) complexed to calcitriol^58^ (PDB code: 1DB1). Blind docking runs including 100 runs each were set up in the following way. Briefly, the target and ligand molecules were equipped with Gasteiger charges using AutoDock Tools (ADT)^57^. All torsion angles of the calcitriol were left free to vary during the minimization. The ERp57 coordinates were kept fixed during the docking simulations. The dimensions of the box were large enough to include the whole protein, and the grid spacing was set to 0.375 Å. Grid searching was performed using the Lamarckian genetic algorithm. The number of energy evaluations was 2.5 × 10^6^ (25 × 10^6^ for focused dockings) and a population size of 200 was applied. All other parameters were set at their default values. All poses of calcitriol were subsequently clustered. Docking runs were started with a random ligand position and orientation. The docking poses were analyzed with ADT and the images were generated with Pymol (The PyMOL Molecular Graphics System, Version 0.99rc6 Schrödinger, LLC)^59^ and ADT.

## Additional Information

**How to cite this article**: Gaucci, E. *et al*. Analysis of the interaction of calcitriol with the disulfide isomerase ERp57. *Sci. Rep*. **6**, 37957; doi: 10.1038/srep37957 (2016).

**Publisher's note:** Springer Nature remains neutral with regard to jurisdictional claims in published maps and institutional affiliations.

## Supplementary Material

Supplementary Information

## Figures and Tables

**Table 1:** Data obtained by calorimetric analysis of ERp57 and ERp57 selected domains in the presence of calcitriol.

	N	Kd	ΔH (cal/mol)	TΔS
a’-ERp57	0.720 ± 0.609	6.47 ± 0.74 × 10^−10^	2.83 ± 0.02 × 10^5^	2.95 × 10^5^
abb’-ERp57	0.483 ± 0.077	1.81 ± 0.13 10^−7^	8.00 ± 0.53 × 10^3^	1.72 × 10^4^
*ERp57 (silib)	—	1.76 ± 0.35 × 10^−9^	5.36 ± 3.00 × 10^4^	**—**

**Table 2:** ERp57 and ERp57 selected domains concentration and ligands amount used in the calorimetric analysis.

	[protein or mutants]	Vitamin D
*abb’*- ERp57	**20 μM**	**100 μM**
*a’*- ERp57	**0.05 μM**	**0.5 μM**
ERp57 (silibinin)	**5 μM (50 μM)**	**10 μM**
