# Supplementary material for: Analysis of the interaction of calcitriol with the disulfide isomerase ERp57
Source: Sci Rep. 2016 Nov 29;6:37957. doi: 10.1038/srep37957 (PMC5126700; doi:10.1038/srep37957)
Supplement: Supplementary Information [file srep37957-s1.doc]

**Supplementary Information**

**Analysis of the interaction of calcitriol with the disulfide isomerase ERp57**

**Elisa Gaucci^1^, Domenico Raimondo^2^**^‡^**, Caterina Grillo^1^, Laura Cervoni^1^, Fabio Altieri^1,3^, Giulio Nittari^1,†^, Margherita Eufemi^1,3^ and Silvia Chichiarelli^1^,***

^1^Department of Biochemical Sciences “A. Rossi Fanelli”, Sapienza University of Rome, Piazzale Aldo Moro 5, 00185, Rome, Italy

^2^Stem Cell Lab - Department of Molecular Medicine - Sapienza Università di Roma - - Viale Regina Elena 324, 00161- Rome - Italy

^3^Istituto Pasteur-Fondazione Cenci Bolognetti, Sapienza University of Rome, Piazzale Aldo Moro 5, 00185, Rome, Italy

^†^Current address: International School of Advanced Studies - University of Camerino - Piazza Cavour 19/f, 62032, Camerino (MC) – Italy

*Corresponding author. Address: Department of Biochemical Sciences“A. Rossi Fanelli”, Sapienza University of Rome, Piazzale Aldo Moro 5, 00185, Rome, Italy
E-mail address: [silvia.chichiarelli@uniroma1.it](mailto:silvia.chichiarelli@uniroma1.it)

^‡^ Co-corresponding author. Address: ^2^Stem Cell Lab - Department of Molecular Medicine Sapienza Università di Roma - Viale Regina Elena 324, 00161- Rome – Italy
E-mail address: [domenico.raimondo@uniroma1.it](mailto:domenico.raimondo@uniroma1.it)

*Purification of recombinant mutant abb’-ERp57*

**Figure 1S**

*
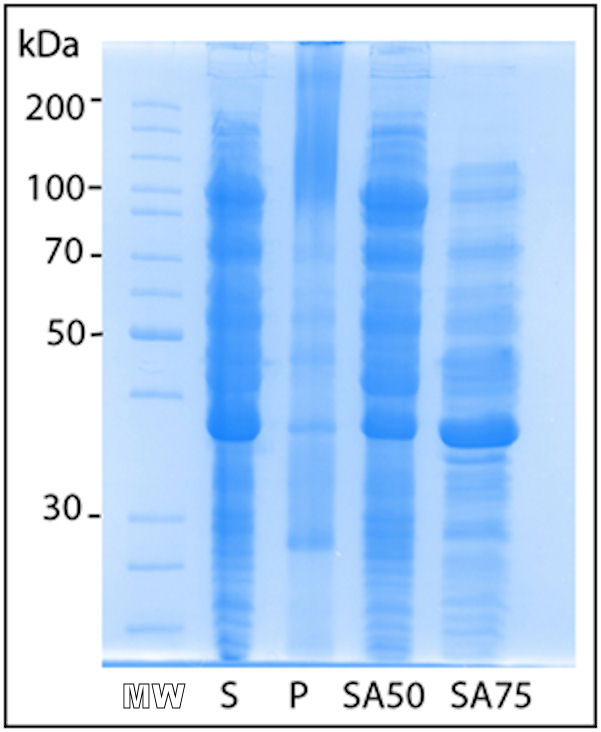
*

**Figure 1S***:* SDS-PAGE of *abb’*-ERp57 mutant. **MW**: Molecular weight; **S**: supernatant after sonication; **P**: pellet after sonication; **SA50**: supernatant after precipitation by 50% ammonium sulfate; **SA75**: pellet supernatant after precipitation by 75% ammonium sulfate.

**Figure 2S**


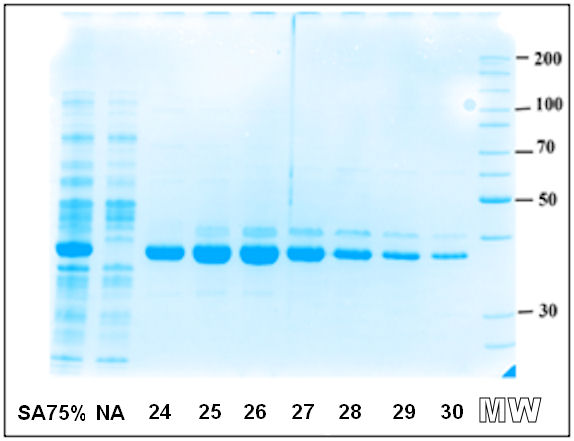


**Figure 2S***:* SDS-PAGE of *abb’*-ERp57 mutant. **SA75**: dialyzed pellet supernatant precipitated by 75% ammonium sulfate; **NA**: non-affine fraction of heparin column; **24-30**: fractions of heparin column with mutant using a narrow NaCl gradient; **MW**: Molecular weight

*Calcitriol interacts with ERp57 and its deletion mutants – fluorimetric assays*

**Figure 3S**


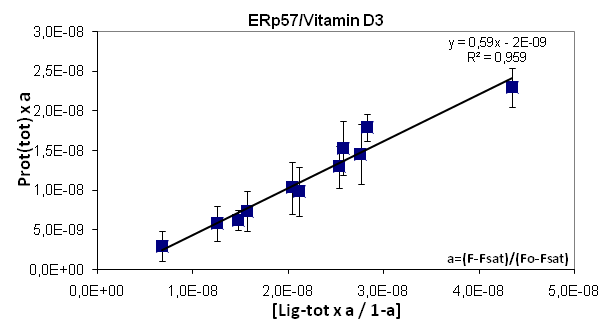


**Figure 3S** Linear least-square plots of Eqn (1), for the titration of ERp57 with calcitriol (Kd ≈ 10^-9^ M and a stoichiometry of ± 1.6 for the number of ligand bound).

**Figure 4S**


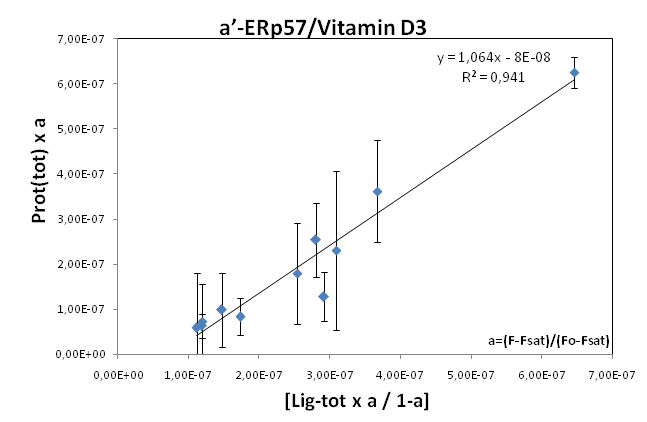


**Figure 4S** Linear least-square plots of Eqn (1), for the titration of *a’*-ERp57 mutant with calcitriol (Kd ~10^-8^ M and a stoichiometry of ± 1.2 for the number of ligand bound).

**Figure 5S**

**
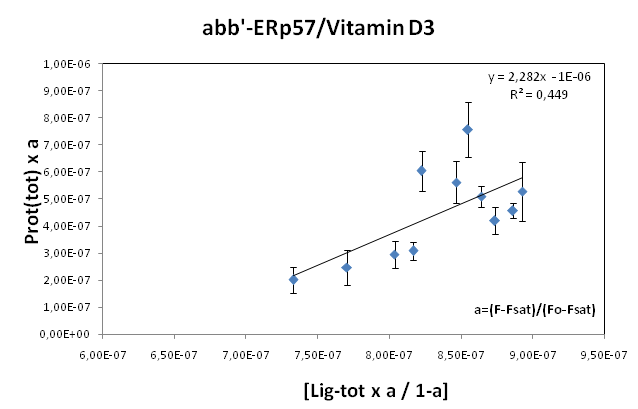
**

**Figure 5S** Linear least-square plots of Eqn (1), for the titration of *abb’*-ERp57 mutant with calcitriol.

*Detection of cavities and hot spots in the ERp57 structure*

By the use of the FTSite server three putative cavities were detected in the ERp57 structure, uploaded as PDB file. One cavity, which is the biggest, is located between the *b* and *b’* domains, while the other two smaller cavities are in the *b* domain (**Fig. 6S**). In **Table 1S**, the residues facing the three cavities are reported, considering their polarity. The structure of ERp57 has been then mapped with the FTMap server; the location of the identified hot spots is shown in **Fig. 7S**. The organic probes clusters are all found in the *b* and *b’* domains, some of them overlapping with the FTSite detected cavities, with the exception of one, which is located in the *a’* domain.

**Figure 6S**

*
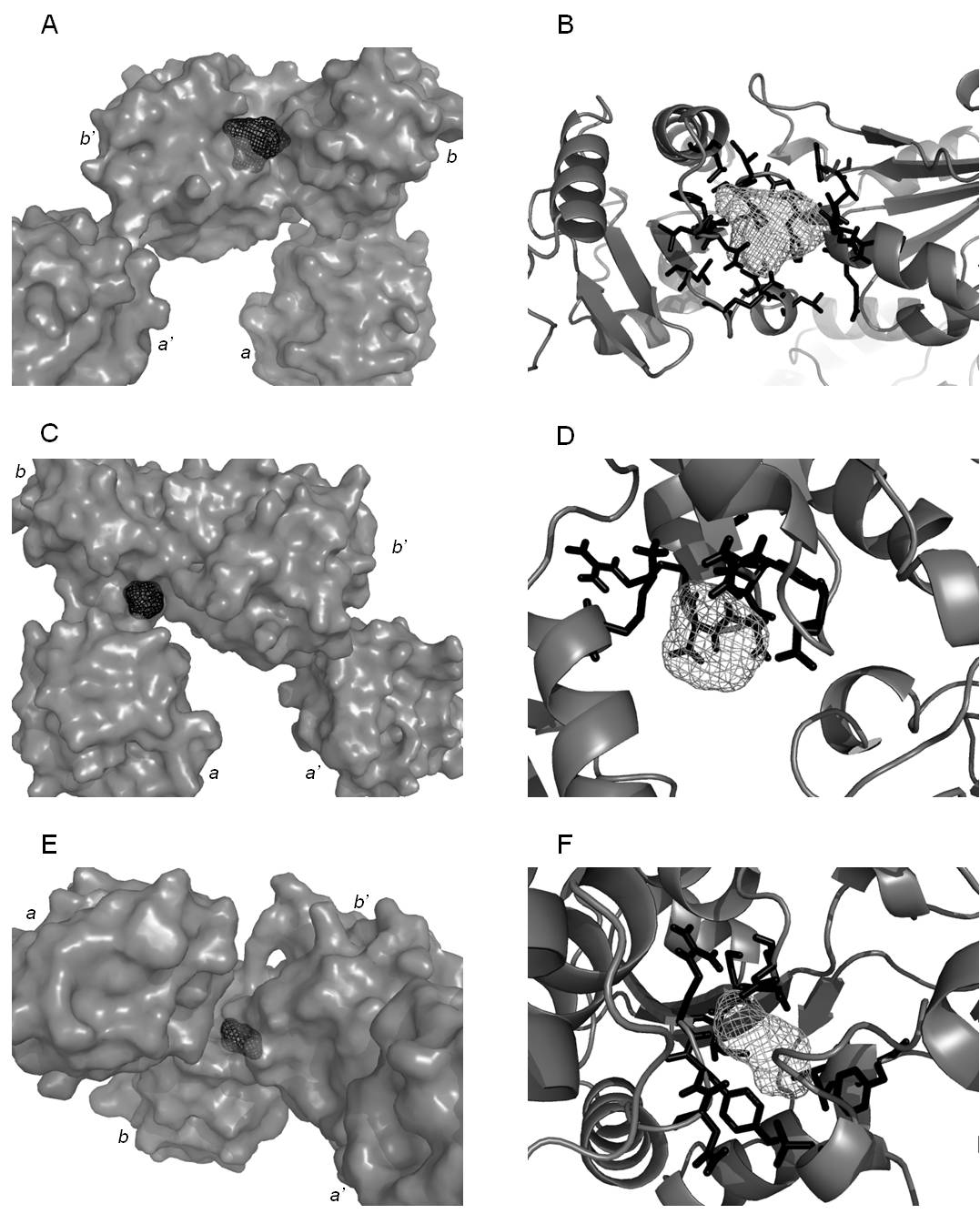
*

**Figure 6S:** Pockets or cavities (in wireframe) detected in the ERp57 structure, with the use of FTSite binding site prediction algorithm. Cavity 1 is located between the *b* and *b’* domains (**A**), while the other two cavities, 2 and 3 (**C** and **E** respectively), are in the *b* domain. On the right column (**B**, **D** and **F**), the detected binding sites are depicted with their surrounding residues (listed in **Table 1S**).

**Table 1S.**

|  | **Hydrophobic aa** | **Polar aa** | **Basic aa** | **Acidic aa** |
| --- | --- | --- | --- | --- |
| **Cavity 1**  (*bb’* domains) | I236, I240, F241, G242, I243, P245, L254, I255, L260, L291, L297, F299, A300 | Q237, N239, C244, N298 | R207, K258 | E238 |
| **Cavity 2**  (*b* domain) | none | N181, Y182 | K130, R179, R183 | D153, D180 |
| **Cavity 3**  (*b* domain) | A154, I156, I240, F241, L254 | S155, N181, Y182 | R183 | D153 |

**Figure 7S**

*
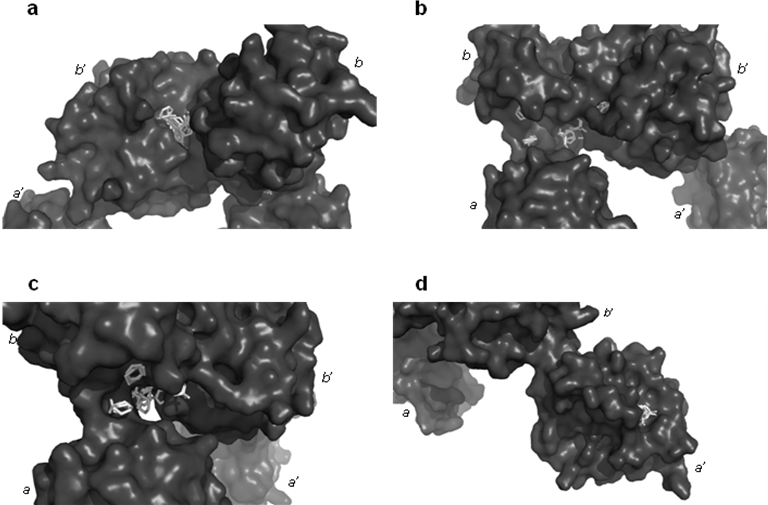
*

**Figure 7S:** Mapping of the ERp57 structure with organic probes, using the server FTMap. The clusters, which are shown in white as sticks, are in the *bb’* domains (**a, b** and **c**), and *a’* domain (**d**).

*Normal mode analysis*

In order to get information about the accessibility of ERp57 binding sites and the possible conformational changes, especially in the hinge regions, the ERp57 structure has been submitted to the elNémo server (http://www.igs.cnrs-mrs.fr/elnemo/index.html) [Suhre, K. & Sanejouand, Y.H. ElNémo: a normal mode web server for protein movement analysis and the generation of templates for molecular replacement. *Nucleic Acids Res.* **32**, W610-614 (2004)], which computes the lowest-frequency modes. In the case of ERp57, five normal modes were obtained, each consisting of 11 frames. The first and last frame were overlapped and the root mean square deviation (RMSD) of the residues surrounding the FTSite detected cavities was calculated (**Table 2S**). The cavities in the five normal modes did not differ much from those in the original ERp57 PDB file. The *a’* resulted the most flexible domain (**Fig. 8S**).

Table 2S.

|  | **Mode 1** | **Mode 2** | **Mode 3** | **Mode 4** | **Mode 5** |
| --- | --- | --- | --- | --- | --- |
| **Cavity 1**  (*bb’* domains) – involved 20 aa | 1.37 Å | 1.34 Å | 1.30 Å | 1.17 Å | 2.07 Å |
| **Cavity 2**  (*b* domain) – involved 7 aa | 1.30 Å | 1.76 Å | 0.68 Å | 1.60 Å | 2.81 Å |
| **Cavity 3**  (*b* domain) – involved 10 aa | 0.88 Å | 1.53 Å | 0.82 Å | 1.23 Å | 1.93 Å |
| ***a’* domain** – involved 104 aa | 4.12 Å | 3.34 Å | 5.28 Å | 1.94 Å | 3.15 Å |

**Figure 8S**


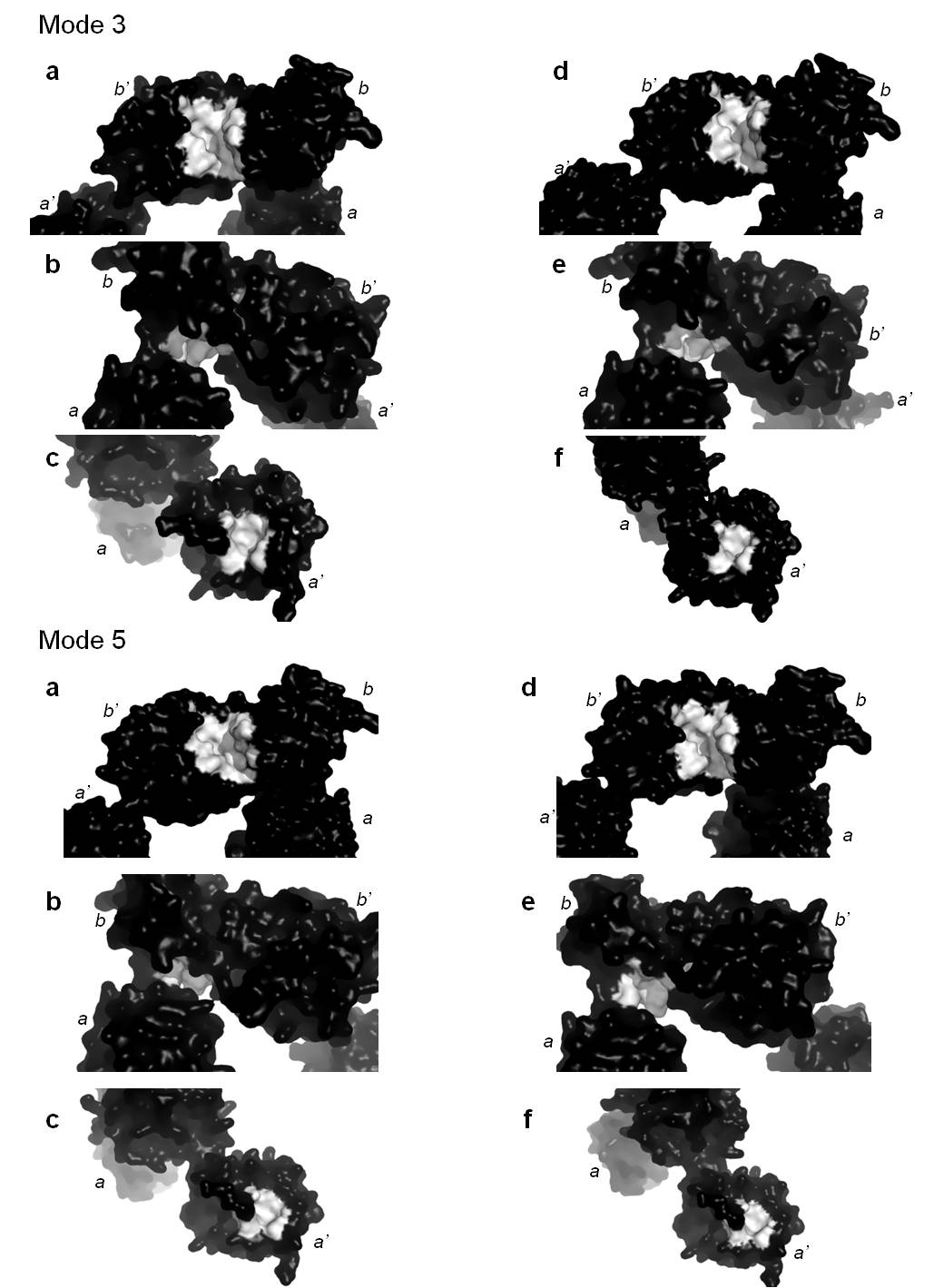


**Figure 8S:** Two of the five lowest-frequency modes computed with the ElNémo server. The first (**a**, **b**, **c**) and last frames (**d**, **e**, **f**) are shown. The surface corresponding to the residues facing the cavities 1 (in **a** and **d**), 2 and 3 (in **b** and **e**) and in the a’ domain (in **c** and **f**) is depicted in white.

**Figure 9S**

**
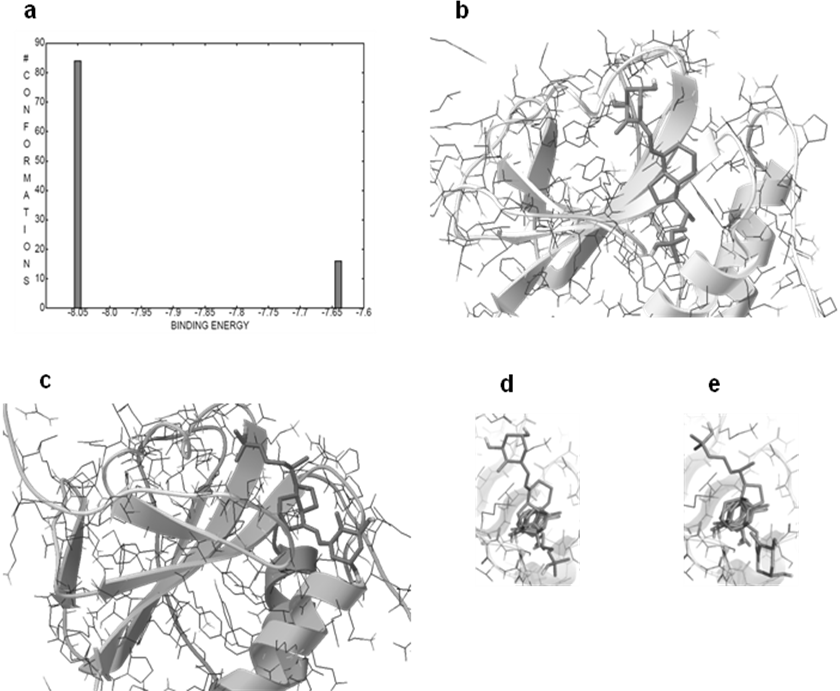
**

**Figure 9S**: **a**: The histogram shows the clustering at 10.0 Å RMSD (root mean square deviation). **b**: the lowest energy conformer is shown in sticks. **c**: the lowest energy conformer of the second cluster is shown in sticks. **d**: superimposition of the lowest energy conformer with the hot spot found by FTMap. **e**: superimposition of the lowest energy conformer of the second cluster with the hot spot found by FTMap.

*The interaction with calcitriol does not inhibit the ERp57 reductase activity*

The effect of calcitriol on the reductase activity of ERp57 has been assessed by evaluating the reduction of FITC-conjugated insulin in a spectrofluorimetric assay. DTT is known to slowly reduce the disulfide bonds of FITC-insulin, and the reaction is robustly enhanced by the addition of ERp57. In order to assess if the binding of calcitriol to recombinant ERp57 could influence the reductase activity of the protein, increasing concentrations of calcitriol were used (0.1, 1, 5, 10 µM) and the emission at 519 nm was followed. It could not be noticed any significant variation of the slope, implying that the interaction of calcitriol with ERp57 does not affect the enzymatic activity of the protein.

**Figure 10S**


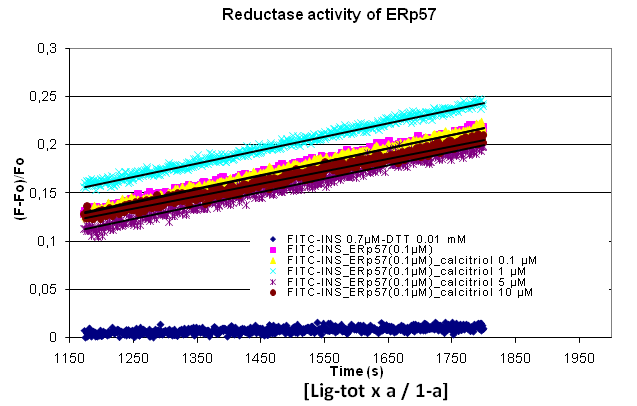


**Figure 10S** Effect of calcitriol on reductase activity of ERp57 performed by fluorescence enhancement of di-FITC-insulin (pH 7.5 and 20°C; 0.01 mM DTT). Emission was measured as described: emission intensity at 519 nm (excitation wavelength, 495 nm).

**Table 3S.**

|  | Slope % | R^2^ | Eqn (trend line) |
| --- | --- | --- | --- |
| FITC-INS_ERp57(0.1µM) | 100 | 0.989 | y = 0,000139x - 0,032925 |
| FITC-INS_ERp57(0.1µM)_calcitriol 0.1 µM | 101 | 0.987 | y = 0,000141x - 0,035944 |
| FITC-INS_ERp57(0.1µM)_calcitriol 1 µM | 100 | 0.988 | y = 0,000139x - 0,006837 |
| FITC-INS_ERp57(0.1µM)_calcitriol 5 µM | 100 | 0.965 | y = 0,000140x - 0,051833 |
| FITC-INS_ERp57(0.1µM)_calcitriol 10 µM | 92 | 0.984 | y = 0,000129x - 0,026764 |
